# Phytochemical Constituents of Propolis Flavonoid, Immunological Enhancement, and Anti-porcine Parvovirus Activities Isolated From Propolis

**DOI:** 10.3389/fvets.2022.857183

**Published:** 2022-04-08

**Authors:** Xia Ma, ZhenHuan Guo, Yana Li, Kun Yang, Xianghui Li, Yonglu Liu, Zhiqiang Shen, Li Zhao, Zhiqiang Zhang

**Affiliations:** ^1^Medicinal Engineering Department, Henan University of Animal Husbandry and Economy, Zhengzhou, China; ^2^Zhengzhou Key Laboratory of Veterinary Immunopharmacology, Henan University of Animal Husbandry and Economy, Zhengzhou, China; ^3^Henan Research Center for Inheritance and Innovation Technology of Classical and Prescriptions of Chinese Veterinary, Zhengzhou, China; ^4^School of Animal Science, Yangtze University, Jingzhou, China; ^5^Binzhou Animal Science and Veterinary Medicine Academy of Shandong Province, Binzhou, China; ^6^College of Pharmacy, Henan University of Chinese Medicine, Zhengzhou, China

**Keywords:** phytochemical constituents, propolis flavonoid, UPLC-Q/TOF-MS/MS, ferulic acid, porcine parvovirus

## Abstract

Propolis is widely used in health preservation and disease healing; it contains many ingredients. The previous study had revealed that the ethanolic or water extracts of propolis have a wide range of efficacy, such as antiviral, immune enhancement, anti-inflammatory, and so on, but its antiviral components and underlying mechanism of action remain unknown. In this study, we investigated the chemical composition, anti-porcine parvovirus (PPV) effectiveness, and immunological enhancement of propolis flavone ethanolic extracts. The chemical composition of propolis flavone was distinguished by ultra-performance liquid chromatography-quadrupole/time-of-flight tandem mass spectrometry analysis. In this study, the presence and characterization of 26 major components were distinguished in negative ionization modes to evaluate the effects of propolis flavonoid used as an adjuvant on the immune response of Landrace–Yorkshire hybrid sows immunized with an inactivated vaccine of PPV. Thirty Landrace-Yorkshire hybrid sows were randomly assigned to one of three groups, and the sows in the adjuvant groups were intramuscularly injected with PPV vaccine with a 2.0-ml propolis flavonoid adjuvant (PA) and oil emulsion adjuvant. After that, serum hemagglutination inhibition antibody titers and specific immunoglobulin (Ig)M and IgG subclasses were measured to evaluate the adjuvant effects of propolis flavonoid on the humoral immune responses, as well as peripheral lymphocyte proliferation activity and serum concentrations of Th1 and Th2 cytokines for cellular immunity. Results indicated an enhancing effect of PA on IgM, interleukins 2 and 4, interferon-γ, and IgG subclass responses. Especially in the effect of improving cellular immune response, the PA was the best. These findings suggested that PA can significantly enhance the immune responses against the PPV vaccine and could be an alternative way to improve PPV vaccination in sows. Furthermore, we screened the PF chemical components to the effectiveness of anti-PPV. Ferulic acid has an excellent anti-PPV effect.

## Introduction

Nature products and natural product derivatives have drawn more attention due to their wide spectrum of biological and pharmaceutical properties. Propolis is a gelatinous solid substance with an aromatic smell, which is collected from plant trunks and flower bracts by bees and mixed with the secretions of their maxillary glands and beeswax (1, 2). The pharmaceutical industries have widely marketed propolis as alternative medicine and as a healthy food in various parts of the world (3). Bioactive constituents isolated from the propolis, flavonoids, terpenes, organic acids, fatty acids, amino acids, enzymes, vitamins, trace elements, and minerals have applications in treating diseases due to their anticancer, anti-inflammatory, antioxidant, antibacterial, antimycotic, antifungal, and immunomodulatory properties (4). So far, more than 500 chemical compounds have been isolated from propolis (5). Also, our previous studies revealed that propolis and its constituents exerted immunological enhancement *in vivo*, as well as the anti-porcine parvovirus (PPV) effect *in vitro*.

PPV is one of the main pathogens that cause swine reproductive disorder and increasing economic losses in the world (6–8). Several studies have pointed out that vaccination is an effective method for controlling this disease (9). The successful vaccination depends on their association with a potent adjuvant, which can increase vaccine immunogenicity. A better adjuvant can activate specific effectors of the immune system, such as cytotoxic or auxiliary T cells (Th1/Th2) and strengthen the humoral and/or cellular immune responses against that antigen (10, 11). On the other hand, a suitable adjuvant should have lower toxicity and adverse effects (12). However, there are some shortages in commonly used adjuvants; for example, oil emulsion adjuvant (OA) can cause inflammation, induration, or necrosis in the local and disseminated granulomas in lungs, liver, kidneys, heart, lymph nodes, and skeletal muscles in rabbits or rats (13). An aluminum salt adjuvant is a widely used adjuvant in human vaccines licensed by the US Food and Drug Administration (14), but it is a poor inducer of Th1 cellular immune response and easy to induce immunoglobulin E antibody response associated with some allergic reactions (15, 16). Thus, the natural product propolis as an immunologic adjuvant has attracted more and more attention due to its minor adverse effects and more pharmacological efficacy.

Most of the studies in the literature have investigated the anti-PPV and immunological enhancement effectiveness of propolis flavonoid (PF) isolated from propolis (17). PF, a kind of ingredient extracted from propolis, as a harmless natural adjuvant, has been used in chickens vaccinated with an activated vaccine, and the results showed that PF could improve the immune-enhancing activity in the humoral and cellular immune response (18). Because the major effectiveness of propolis is derived from the active ingredient, the anti-PPV and immunological enhancement effect are highly dependent on its extraction method and the content of the active ingredients of PF. Ethanol-extracted propolis is still the main method for PFs. However, still little is known regarding the anti-PPV effect of PF compounds, such as ferulic acid, quercetin, chrysin, and so on.

Thus, in this study, an ultra-performance liquid chromatography-quadrupole/time-of-flight mass spectrometry (UPLC-Q/TOF-MS) is used to distinguish and exhibit the standard chemical map of PF. In addition, this study aimed to evaluate the adjuvant effect and characteristics of PF on the humoral and cellular immune response of immunized pigs and screen out good anti-PPV PF compounds.

## Materials and Methods

### Propolis Sample Collection, Extraction, and Processing

Propolis samples were collected in September of 2018 in Liaocheng Apiculture Research Institute, Shandong province, China. The ethanolic extract was prepared as reported by Almeida et al. (19). Ten grams of the powder was mixed with 100 ml of 75% ethanol in a sealed container protected from light (to avoid loss of volatile and photosensitive compounds) under agitation in a water bath at 70°C for 30 min. After extraction, the mixture was filtered. The filtrated liquid was combined with the original liquid. The ethanol was recovered by rotary evaporation and left overnight to obtain the crude propolis extract in the upper layer and the crude propolis extract in the lower layer.

### Mass Spectrum Analysis and Verification of Methodology

Propolis flavone sample analysis was managed and consisted of SIL-30SD autosampler, Shimadzu UPLC (Kyoto, Japan), CTO-30A column oven, an LC-30AD Binary liquid pump, DGU-20A5R On-Line Solvent Degasser, AB SCIEX Triple TOF 5600+ system, and ESI source. Chromatographic conditions were as follows: performed on a C18 reversed-phase LC column (Agilent ZorBax SB-C18 50 × 2.1 mm, 1.8 mm), and the column temperature was maintained at 25°C. The mobile phase consisted of 0.1% (v/v) formic acid water (solvent A) and acetonitrile (solvent B) using a gradient program as follows. Gradient elution program was as follows: 5–25% B, 0–1 min; 25–30% B, 1–4 min; 30–55% B, 4–12 min; 55–70% B, 12–1 8min; 70–100% B, 18–25 min; 100–5% B, 25–28 min; flow rate: 0.3 ml/min; column temperature: 35°C; injection volume: 1 μl. Mass spectrometry conditions are as follows: ESI source and data collection in positive and negative ion modes. The source parameters were set as follows: ion spray voltage floating: +4,500/−4,500; declustering potential: +60/−60 V; source temperature: 550°C; the atomizing gas is N2, curtain gas: 35 psi; gas1 (nebulizer gas): 55 psi; gas2 (heater gas): 55 psi; collision energy: +35/−35 e V; using tandem mass spectrometry secondary mode: The mass spectrometry ion scanning range was m/z 100–2,000. The tandem mass spectrometry ion scanning range was m/z 50–1,000; turn on dynamic background subtraction.

### Identification of Chemical Constituents

The ultra-high-performance liquid chromatography (UHPLC)-Q/TOF-MS data of all of the studied samples were analyzed using MarkerView and PeakView software (AB Sciex, Massachusetts, USA) for the purpose of identifying PF chemical constituents.

### Preparation of Adjuvant and Vaccine

PF was prepared in our laboratory in a final purity of 925 mg·g^−1^. The PA was prepared as previously described. The PF was dissolved in phosphate-buffered saline (PBS, pH 6.2), in a final concentration of 40 mg·ml^−1^. The China Institute of Veterinary Drug Control supplied PPV. After propagating *in vitro* in PK-15 cell cultures, the virus that contained 2,048 hemagglutination units (HAU)·ml^−1^ was inactivated with formaldehyde and stirred 24 h at 37°C. The inactivated virus that contained 512 HAU·ml^−1^ was used as a vaccine antigen. Three adjuvant vaccines containing PA or OA were prepared by Lvdu Veterinary Biologicals Co. Ltd., Binzhou, China. Their virus contents were the same.

### Animals, Housing, and Treatment

Thirty Landrace-Yorkshire hybrid sows (aged from 198 to 204 days, and the average weight is 64.5 kg) were randomly assigned to one of three groups, receiving an intramuscular injection of PPV vaccine with 2.0-ml PA, OA, or physiological saline as the blank control group (BC). The animals were kept in 10 pigpens divided equally under standard conditions in the Experimental Animal Center of Binzhou Animal Science and Veterinary Medicine Academy, Shandong province [no. SYXK (lu) 20110066]. They were maintained in an air-conditioned room with light from 06:00 to 18:00 h. The room temperature (24 ± 3°C) and humidity were controlled automatically. They were fed with water and food *ad libitum*. All procedures related to the animals and their care conformed to the internationally accepted principles as found in the Guidelines for Keeping Experimental Animals issued by the government of China. The antibody against PPV was negative before the experiment.

Before vaccination and on days 7, 14, 21, 28, 35, and 49 after vaccination, the blood of six pigs randomly from each group was sampled for determination of serum hemagglutination inhibition (HI) antibody titer of PPV by micro-method dynamically and continuously. On days 7, 14, 21, and 35 after vaccination, the blood of four pigs randomly from each group was sampled for examination of lymphocyte proliferation by methyl thiazolyl tetrazolium (MTT) assay and analyzing specific immunoglobulin (Ig)M and IgG1, IgG2, IgG3, IgG4, interleukin (IL)-2, IL-4, and interferon-γ (IFN-γ) in serum by enzyme-linked immunosorbent assay (ELISA) kit.

### Hemagglutination Inhibition Antibody Titer of Porcine Parvovirus Determination

Blood samples (1.0 ml per pig) from the ear vein were drawn into Eppendorf tubes and allowed to clot at 37°C for 1 h. Serum was separated by centrifugation for the determination of HI antibody. Briefly, twofold serial dilution of 50-μl serum, after inactivated at 56°C for 30 min, was made in a 96-well V-shaped bottom microtiter plate containing 50 μl of calcium/magnesium-free PBS in all wells, then 50 μl of PPV antigen (4 HA units) was added into all wells except for the last row, which served as the controls. Serum dilutions ranged from 1:2 to 1:2,048. The antigen–serum mixture was incubated at 37°C for 10 min. Fifty microlitres of 1% rooster erythrocytes suspension was added into each well and re-incubated for 30 min. A positive serum, a negative serum, erythrocytes, and antigens were also included. The highest dilution of serum causing complete inhibition was considered the endpoint. The geometric mean titer was expressed as reciprocal log2 values of the highest dilution that displayed HI antibody titer.

### Peripheral Lymphocyte Proliferation Assay

The blood sample from the precaval vein was diluted in Hanks' solution. After centrifugation (800 × *g* at 4°C for 10 min), the cells were washed three times with PBS and resuspended in RPMI-1640. The cells were counted with a hemocytometer by trypan blue dye exclusion technique, and their viability exceeded 95%. Briefly, lymphocytes at 5.0 × 106 cell·ml^−1^ were seeded into a 96-well flat-bottom microtiter plate, 100 μl each well, then Con A (final concentration 5 μg·ml^−1^) or medium was added. The plates were incubated in a humid atmosphere with 5% CO_2_ at 37°C for 48 h. All the tests were carried out in quadruplication. The cell proliferation was evaluated by MTT methods. Briefly, 30 μl of MTT solution (5 mg·ml^−1^) was added to each well at 4 h before the end of incubation. The plates were centrifuged (1,000 × *g*, 5 min) at room temperature, and MTT was removed carefully. After 150-μl dimethyl sulfoxide was added into each well, the plates were shaken for 5 min to dissolve the formazan crystals completely, and the absorbance at 570 nm (A570 value) was determined by ELISA reader (BioTek Instruments Inc.) as the index of lymphocyte proliferation.

### Cytokine and Immunoglobulin Determination

Serum concentrations of cytokines (including IL-2, IFN-γ, and IL-4) and specific IgM and IgG subclasses were assayed by ELISA according to the manufacturer's instructions. Briefly, a 96-well flat-bottom microtiter plate (Nunc, USA) was coated with a capture antibody specific for each cytokine. The plate was washed and blocked before 10 μl of the serum, and serially diluted specific standards were added to the respective wells. After a series of washing, the captured cytokine was detected using the specific conjugated detection antibody. The substrate reagent was added into each well, and, after color development, the plate was read at 450 nm using an ELISA reader (BioTek Instruments Inc.). Cytokine concentrations and IgM and IgG subclasses were accomplished by a standard curve plotting the A450 value against each dilution of the standard concentration.

### Screening of Flavonoids in Propolis Against Porcine Parvovirus *in vitro*

PK-15 cells (presented by Binzhou Animal Husbandry and Veterinary Research Institute of Shandong province) were cultured in DMEM medium containing 10% fetal calf serum (South American origin) in a 5% CO_2_ cell incubator at 37°C, with 0.25% trypsin (0.02% ethylenediaminetetraacetic acid) digestion passage.

In PK-15 cells with trypsin digestion, 1 × 10^5^/ml was vaccinated in 96 well plates, 100 μl per hole, under the condition of 37°C and 5% CO_2_ training for 24 h; the supernatant was abandoned, and PBS washing was done, except for the blank group, which added DMEM, containing the rest of the holes to join with a multiplicity of infection = 1.0 PPV cultures of 100 μl, training after 4 h. Then, the supernatant was abandoned, except the blank group and model group with DMEM; in the remaining wells, the 100-μl medium was added with a 50-μM concentration of galangin, kaolin, quercetin, ferulic acid, aspen, and apigenin, and four replicates were set for each drug. After 48 h, the supernatant was discarded, and a 10-μl CCK-8 medium was added to a 100-μl DMEM medium per well for a 30-min culture, and the absorbance value of CCK-8 was detected.

### Statistical Analysis

Data analysis was performed with SPSS 19.0 software (SPSS Inc., Chicago, IL, USA). Differences in mean values among the polysaccharide and control groups were analyzed by one-way analysis of variance. The data were expressed as mean ± standard error. Values of P < 0.05 were considered to be statistically significant.

## Results

### Identification of Active Components of Alcohol Extract by Ultra-High-Performance Liquid Chromatography-Quadrupole/Time-of-Flight Mass Spectrometry

**Figure 1:**
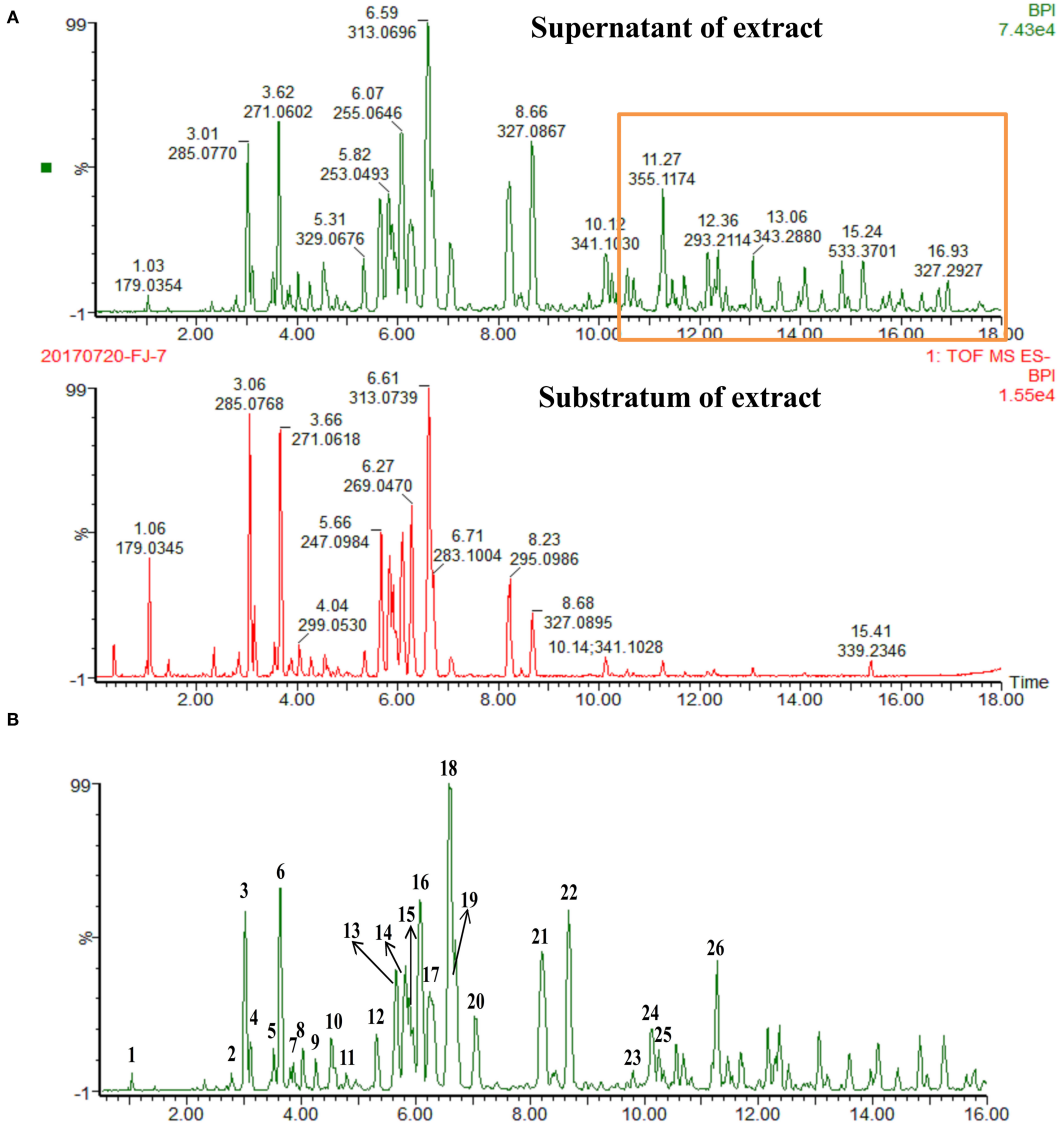
UHPLC-Q/TOF-MS chemical fingerprints (TIC chromatography) of propolis extract. **(A)** Superstratum and substratum of propolis ethanol extract. **(B)** (1) Caffeic acid, (2) Quercetin, (3) 5-Methoxypinobanksin, (4) 3-Methoxyquercetin, (5) Apigenin, (6) Pinobanksin, (7) Isorhamnetin, (8) 5-Methoxyluteolin, (9) 5,7-Dimethoxyquercetin, (10) 5-Methoxygalangin, (11) 7-Methoxyquercetin, (12) 7-Methoxyquercetin-X-methyl ester, (13) Isoprene caffeic acid ester, (14) Chrysin, (15) Caffeic acid benzyl ester, (16) Pinocembrin, (17) Galangin, (18) 3-O-Acetylpinobanksin, (19) Caffeic acid phenethyl ester, (20). Methoxychrysin, (21) Cinnamate caffeic acid, (22) Pinobanksin-3-O- propanoate, (23) P-Cinnamyl coumarate, (24) Pinobanksin-3-O-butyrate, (25) Pinobanksin-3-O-pentenoic acid ester, (26) Pinobanksin-3-O-valerate.

The crude total PFs were ~80% in the alcohol extraction. According to the analytical results for the chemical constituents (as shown in Figure 1A), the different components in the supernatant and substratum of propolis extract are mainly small polar components. We analyzed the chemical fingerprint (UHPLC-Q/TOF-MS) of PF (Figure 1B). The analysis in negative ionization modes revealed the presence and characterization of 26 major components (Table 1).

**Table 1:** Identification of compounds in ethanolic crude extract from propolis of Melipona quadrifasciata by UPLC-MS analysis.

**Peak No**.	**Rt (min)**	**Tentative identification**	**Chemical** **formula**	**[M-H]- (m/z)**		
				**Mean measured mass (Da)**	**Theoretical exact mass (Da)**	**Mass accuracy (ppm)**
1	1.03	Caffeic acid	C9H8O4	179.0345	179.0344	0.6
2	2.78	Quercetin	C15H10O7	301.0348	301.0350	0.7
3	3.01	5-Methoxypinobanksin	C16H14O5	285.0768	285.0763	1.8
4	3.10	3-Methoxyquercetin	C16H12O7	315.0507	315.0505	0.6
5	3.51	Apigenin	C15H10O5	269.0455	269.0450	1.9
6	3.62	Pinobanksin	C15H12O5	271.0602	271.0606	−1.5
7	3.86	Isorhamnetin	C16H12O7	315.0499	315.0505	−1.9
8	4.02	5-Methoxyluteolin	C16H12O6	299.0541	299.0556	−5.0
9	4.25	5,7-Dimethoxyquercetin	C17H14O7	329.0666	329.0661	1.5
10	4.52	5-Methoxygalangin	C16H12O5	283.0610	283.0606	1.4
11	4.78	7-Methoxyquercetin	C16H12O7	315.0497	315.0505	−2.5
12	5.31	7-Methoxyquercetin-X- methyl ester	C17H14O7	329.0653	329.0661	−2.4
13	5.63	Isoprene caffeic acid ester	C14H16O4	247.0974	247.0970	1.6
14	5.82	Chrysin	C15H10O4	253.0493	253.0501	−3.2
15	5.89	Caffeic acid benzyl ester	C16H14O4	269.0825	269.0814	4.1
16	6.07	Pinocembrin	C15H12O4	255.0646	255.0657	−4.3
17	6.24	Galangin	C15H10O5	269.0457	269.0450	2.6
18	6.59	3-O-Acetylpinobanksin	C17H14O6	313.0696	313.0712	−5.1
19	6.69	Caffeic acid phenethyl ester	C17H16O4	283.0966	283.0970	−1.4
20	7.04	Methoxychrysin	C16H12O5	283.0602	283.0606	−1.4
21	8.20	Cinnamate caffeic acid	C18H16O4	295.0970	295.0970	0.0
22	8.66	Pinobanksin-3-O- propanoate	C18H16O6	327.0867	327.0869	−0.6
23	9.80	P-Cinnamyl coumarate	C18H16O3	279.1031	279.1021	3.6
24	10.12	Pinobanksin-3-O-butyrate	C19H18O6	341.1030	341.1025	1.5
25	10.25	Pinobanksin-3-O- Pentenoic acid ester	C20H18O6	353.1035	353.1025	2.8
26	11.27	Pinobanksin-3-O-Valerate	C20H20O6	355.1174	355.1182	−2.3

### Identification and Characterization of Propolis Flavonoids

**Figure 2:**
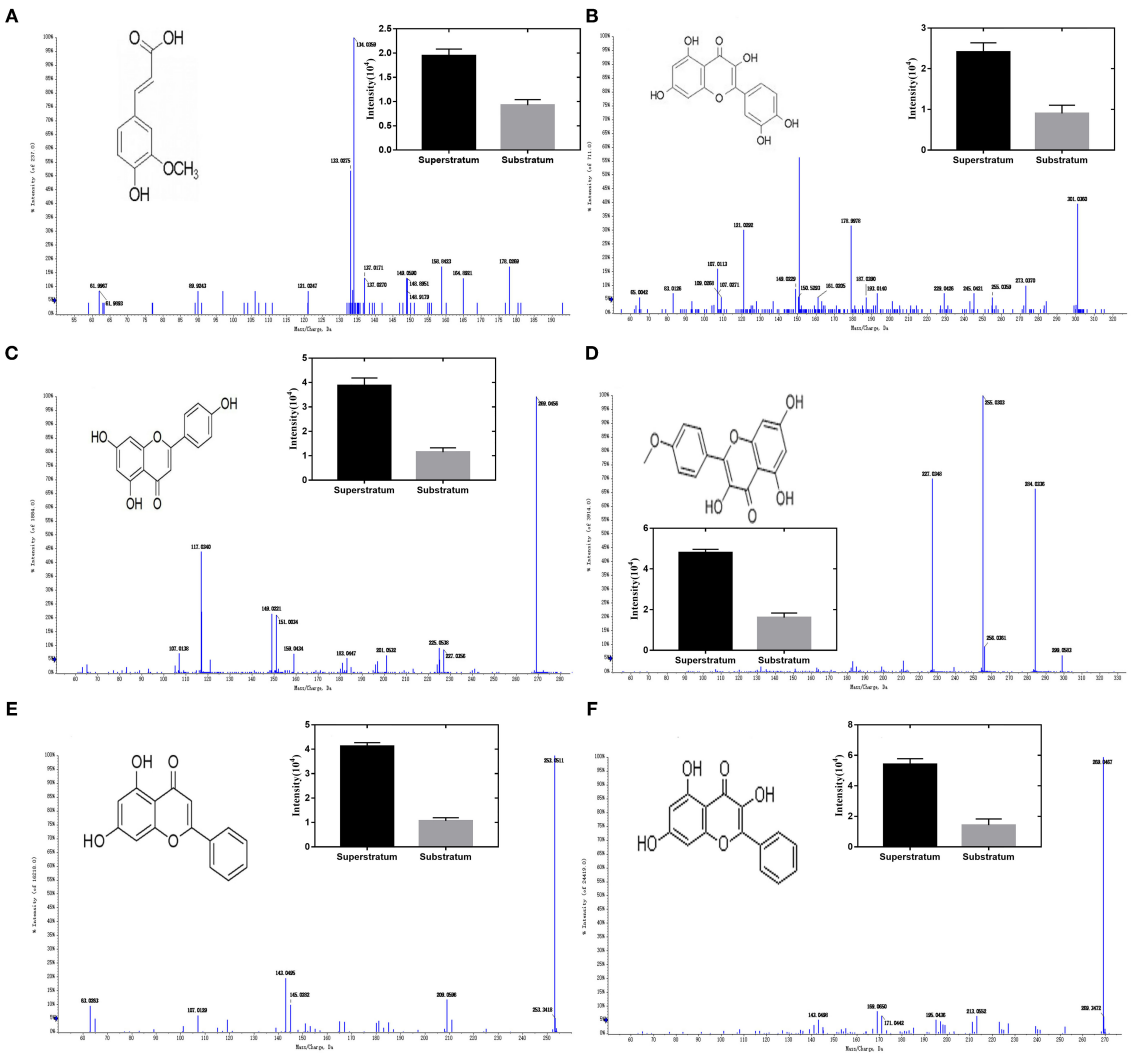
Ion chromatogram of flavonoids in propolis. **(A)** Ferulic acid, **(B)** quercetin, **(C)** apigenin, **(D)** kaempferol, **(E)** chrysin, and **(F)** galangal.

Figure 2A results show that the molecular composition of the compound was C10H10O4. In the anion mode, m/z 194 [M-H]-, typical fragment ion peaks such as 178 and 164 were found in the secondary mass spectrogram, and m/z 194 lost OCH3 to obtain m/z 178, which was speculated to be ferulic acid by comparing the mass spectrogram information with the reference substance.

Figure 2B results show that the molecular composition of the compound was C15H10O7. In the anion mode, m/z 301 [M-H]-, the secondary mass spectrogram showed typical fragment ion peaks such as 273, 179, 151, and 107, m/z 301 lost CO to obtain m/z 273, aglycone RDA reaction produced m/z179, 151, and 107, and compound B was speculated to be Quercetin by comparing the mass spectrogram information with the reference substance.

Figure 2C results show that the molecular composition of the compound was C15H10O5. In the anion mode, typical fragment ion peaks such as 151 and 117 were found in the secondary mass spectrogram of m/z 269[M-H]-, and compared with the mass spectrogram of the reference substance, compound C was speculated to be apigenin.

Figure 2D results show that the molecular composition of the compound was C15H10O6. In the anion mode, typical fragment ion peaks such as m/z 284 [M-H]- and 255 and 227 were found in the secondary mass spectrogram. m/z 284 lost COH to get m/z 255, whereas m/z 255 lost CO to get m/z 227. By comparing the mass spectrogram information with the reference substance, compound D was speculated to be kaempferol.

Figure 2E results show that the molecular composition of the compound was C15H10O4. In the anion mode, m/z 253 [M-H]-, secondary mass spectrometry 143, 63, and other typical fragment ion peaks were found. By comparing the mass spectrometry information with the reference substance, it was speculated that compound E was chrysin.

Figure 2F results show that the molecular composition of the compound was C15H10O5. In the anion mode, the ion peaks of typical fragments such as 213, 171, and 169 were found in the secondary mass spectrogram of m/z 269 [M-H]-, and the mass spectrogram information was compared with the reference substance, suggesting that compound E was galangal.

### Serum Immunoglobulin M and G Subclasses Detected by Enzyme-Linked Immunosorbent Assay

**Figure 3:**
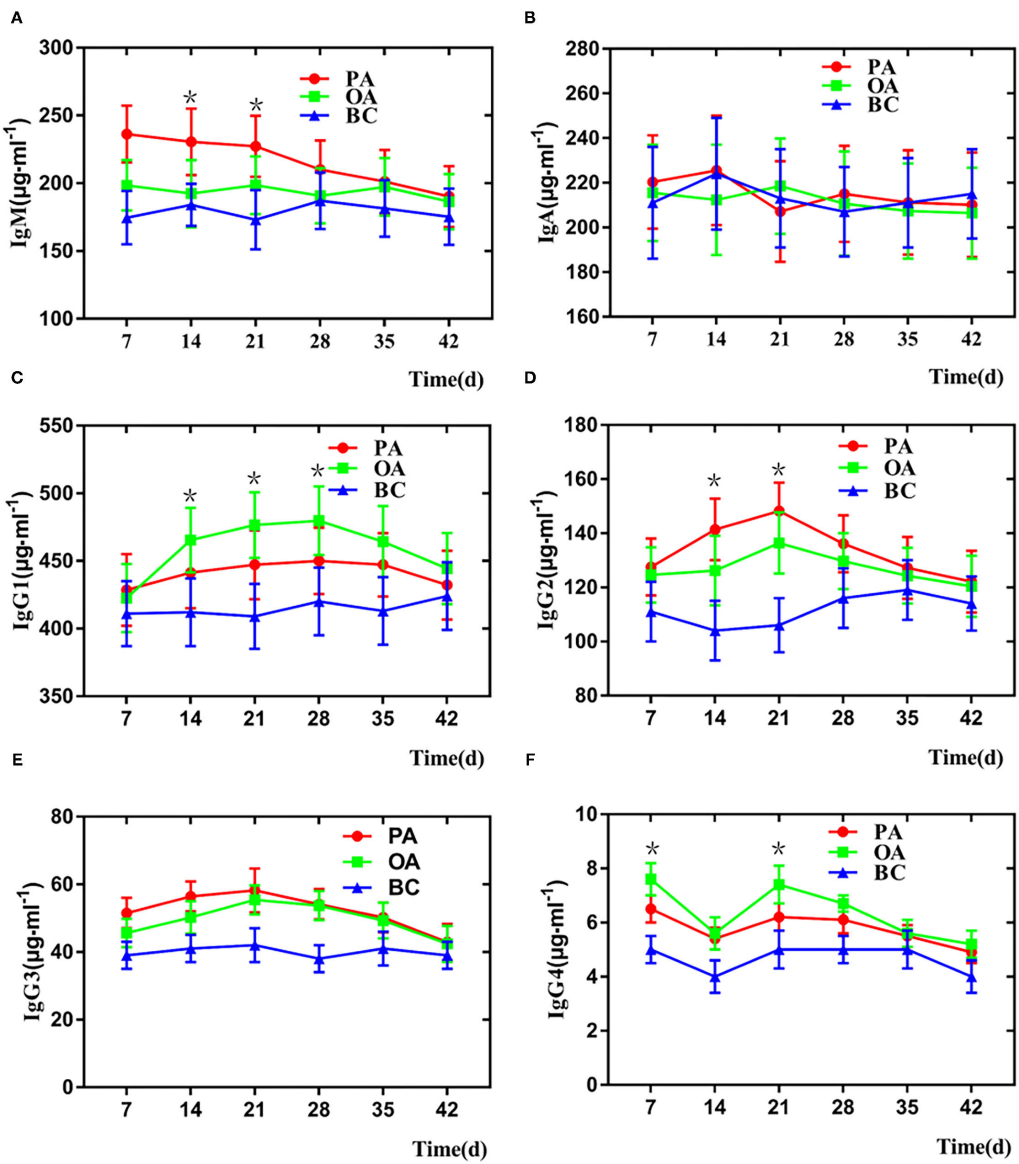
Change of serum IgM and IgG in each group. **(A)** IgM, **(B)** IgA, **(C)** IgG1, **(D)** IgG2, **(E)** IgG3, and **(F)** IgG4. *There is a significant difference compared with BC group.

As illustrated in Figure 3A, on days 7 and 35 after vaccination, the IgM level of the PA group was higher than those of other groups, whereas on days 14 and 21 after vaccination, the IgM level of the PA group only was numerically higher than the OA group and higher than that of the BC group. In addition, the IgA serotype is a monomer, and the immune function is weak. The IgA level of all groups has no significant difference due to the sample being the serum (Figure 3B). IgG1 levels in the two adjuvant groups were higher than that of the BC group when the samples were collected after immunization. On days 14, 21, and 28 after vaccination, the IgG1 levels of the PA group were significantly higher than those of the BC group (Figure 3C). PA triggered a stronger IgG2 level than the OA and BC groups on days 14 and 21, respectively (Figure 3D). The IgG3 level was higher in the pigs of the PA group than that of the pigs immunized with the OA group. However, no significant difference was observed for the responses of IgG3 between the OA and BC groups (Figure 3E). As illustrated in Figure 3F, on days 7 and 35 after vaccination, the IgG4 level of the PA group was higher than those of other groups, whereas on days 7 and 21 after vaccination, the IgG4 level of the PA group was only numerically higher than the OA group and higher than that of the BC group.

### Propolis Flavonoid Promoted the Activity of Peripheral Lymphocyte Proliferation

The lymphocyte proliferation activities are shown in Table 2. In all adjuvant groups, the peak values of lymphocyte proliferation activity were on day 7 after vaccination. The A570 values in the PA group were significantly highest and significantly higher than the OA group from days 7 to 21. On day 35, the A570 values in the two adjuvant groups were significantly higher than that of the BC group.

**Table 2:** Changes in lymphocyte proliferation of blood (A570 value).

**Group**	**D_**7**_**	**D_**14**_**	**D_**21**_**	**D_**35**_**
PA	0.158 ± 0.015^a^	0.140 ± 0.013^a^	0.142 ± 0.009^a^	0.133 ± 0.010^a^
OA	0.120 ± 0.010^b^	0.127 ± 0.015^b^	0.137 ± 0.012^b^	0.130 ± 0.027^a^
BC	0.100 ± 0.021^c^	0.108 ± 0.012^c^	0.120 ± 0.013^c^	0.112 ± 0.012^b^

*PA, propolis adjuvant; OA, oil emulsion adjuvant; BC, Blank control, the same as follows*.

^a−c^*Data within a column without the same superscripts differ significantly (p < 0.05)*.

### Serum Antibody Titer

The serum HI antibody titers of each group are shown in Table 3. As compared with the BC group, the antibody titers in all vaccination groups increased (*p* < 0.05). On days 21, 28, 35, and 42, the antibody titers in the PA group were significantly lower than those in the OA group.

**Table 3:** Dynamic variation of HI antibody titer after vaccination (log2).

**Group**	**D_**0**_**	**D_**7**_**	**D_**14**_**	**D_**21**_**	**D_**28**_**	**D_**35**_**	**D_**49**_**
PA	0 ± 0^a^	4.5 ± 0.4^a^	7.4 ± 0.5^a^	7.5 ± 0.4^b^	7.8 ± 0.7^b^	7.3 ± 0.5^b^	6.5 ± 0.5^b^
OA	0 ± 0^a^	4.3 ± 0.5^a^	7.3 ± 0.5^a^	8.5 ± 0.6^a^	8.8 ± 0.5^a^	8.3 ± 0.7^a^	7.5 ± 0.7^a^
BC	0 ± 0^a^	0 ± 0^c^	0 ± 0^c^	0 ± 0^c^	0 ± 0^c^	0 ± 0^c^	0 ± 0^c^

^a−c^*Data within a column without the same superscripts differ significantly (p < 0.05)*.

### Serum Cytokine Level Detected by Enzyme-Linked Immunosorbent Assays

**Figure 4:**
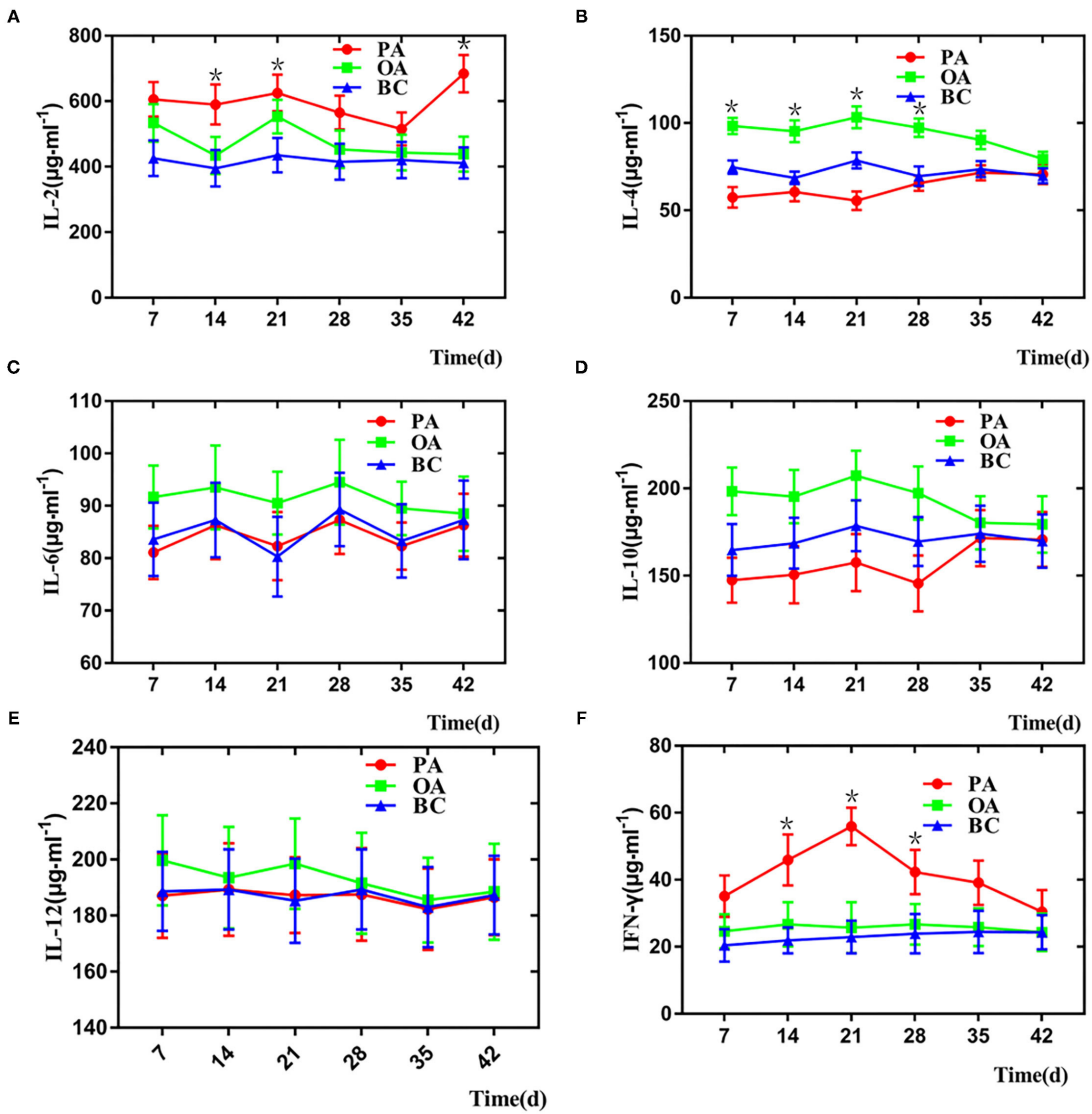
Change of serum cytokine in each group. **(A)** IL-2, **(B)** IL-4, **(C)** IL-6, **(D)** IL-10, **(E)** IL-12, and **(F)** IFN-γ. *There is a significant difference compared with BC group.

Serum cytokine levels are listed in Figure 4. On days 14, 21, and 28, the serum IL-2, IL-4, and IFN- γ levels in two adjuvant groups were significantly higher when compared with those in the BC group (Figures 4A,B,F). Interestingly, the serum IL-6, IL-10, IL-12 levels in the two groups are not significantly higher when compared with those in the BC group (Figures 4C–E). IL-6 and IL-12 could promote the expression level of tumor necrosis factor-α. In addition, despite this, we think PF could be an immune enhancement *in vivo*.

### Screening of Flavonoids in Propolis Against Porcine Parvovirus *in vitro*

**Figure 5:**
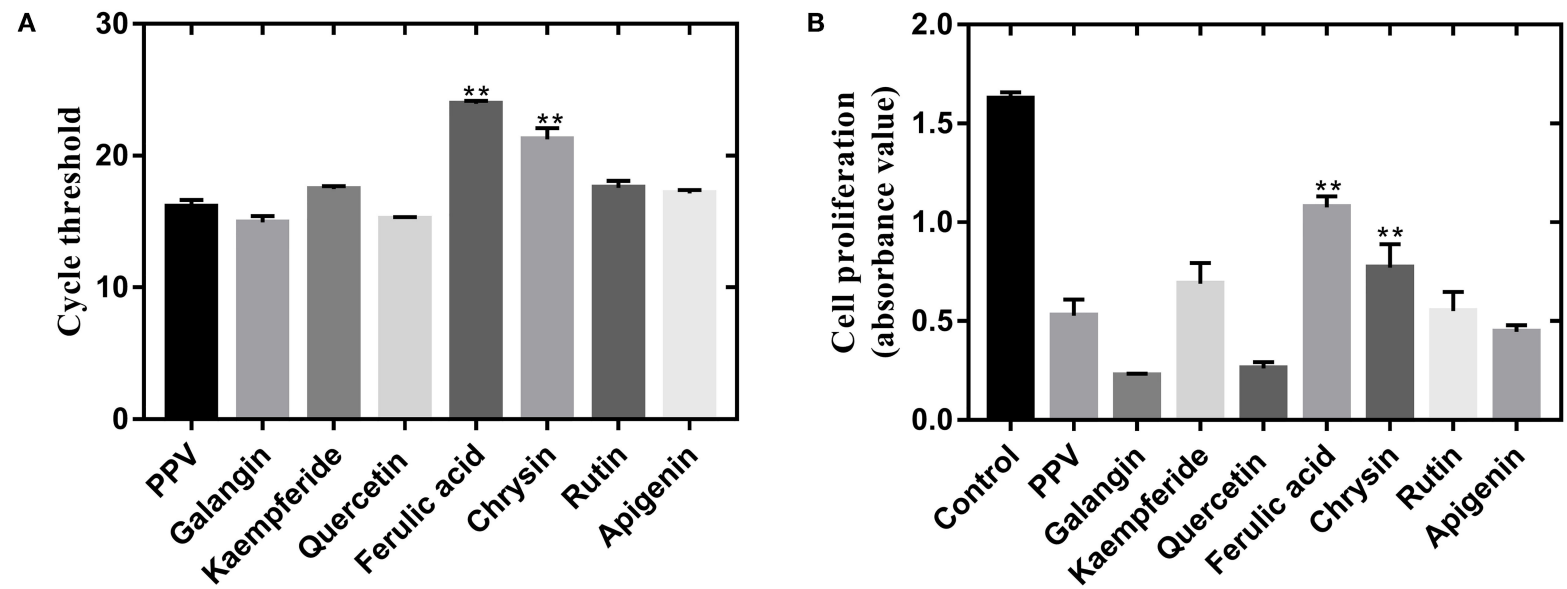
PF isolated from propolis inhibited PPV-induced cell death of PK-15 cells. **(A)** Effect of PF treatment on PPV replication. Virus replication ability was detected by real-time polymerase chain reaction. **(B)** Cell proliferation of PPV-infected PK-15 cells after PF treatment. Cell proliferation was measured by CCK-8 assay. **There is a significant difference compared with PPV group.

As shown in Figures 5A,B, ferulic acid, chrysin, kaempferol, and galangal had a good inhibitory effect on PPV-induced PK-15 cells, but ferulic acid and chrysin had the best effect.

## Discussion

Propolis is an important medicinal material with a variety of pharmacological activities. The chemical analysis of propolis alcohol extract revealed the presence of flavonoids compounds. The traditional method of extracting and purifying traditional Chinese medicine extract is done first, and then, identifying the structure by nuclear magnetic resonance after the monomer is obtained is complicated and requires a large amount of work. UPLC-MS techniques combined with HPLC rapid high separation ability and high sensitivity and high accuracy of high-resolution mass spectrometry, to avoid the disadvantages of traditional component separation analysis method, not only can do a fast qualitative analysis of complex components but also has the characteristics of simple operation, a large amount of information, and saving solvent, thus increasing in the study of Chinese herbal medicine composition.

The current study revealed that PF from propolis had antivirus activity and immune enhancement and antioxidant activity. PF enhances immune responses in various ways, such as the formation of the immunostimulating complex and activation of helper and cytotoxic T cells. In addition to stimulating the humoral immune response, PF also increased cellular immune responses (20, 21). In this study, PF was varied regarding their adjuvant effects on the immune responses that stimulated the immune system cells to produce cytokines. As shown in Figure 4, PF significantly increased the production of both Th2 cytokines IL-4 and Th1 cytokines IL-2 and IFN-γ, which suggested that PF simultaneously elicited a Th1 and Th2 immune response. These findings are consistent with Blonska's study, which demonstrated that PF could enhance the activity of T lymphocytes, promoting the secretion of some cytokines, such as IL-2 and tumor necrosis factor-α, thus improving the immune function of the organism (22).

Th1 activation contributes to cell-mediated immunity, whereas Th2 activation favors the humoral immune response (23). Th1/Th2 balance is a prerequisite for the functionality of the immune system against infections. PF immunomodulatory action has been widely investigated lately, both *in vitro* and *in vivo* (24). PF has been suggested to be a promising adjuvant substance in duck-inactivated vaccines (25). Important functional properties of immune cells are their capability to synthesize and secrete soluble polypeptide factors referred to as cytokines. Most cytokines are secreted and then bind to specific receptors on the surface of target cells. Upon binding, they act to regulate growth and/or differentiation and to optimize the immune response. Using a Polish sample, Ansorge (26) found that propolis has immunoregulatory effects that may be mediated by Erk2 MAP kinase signals that promote cellular growth. Orsolic suggested that propolis stimulated macrophages, thus influencing specific and nonspecific immune defense mechanisms. Activation of macrophages is important for the modulating property of tested compounds, as it leads to the production of factors regulating activities of B and T cells. During a T cell-dependent immune response, there is a progressive change in the predominant immunoglobulin class of the specific antibody produced. This change, isotype switch, is influenced by T cells and their cytokines. IL-4 preferentially switches activated B cells to the IgG1 isotype (Th2 type immune response); IFN-γ enhances IgG2 and IgG3 responses (Th1 type) (27, 28).

In summary, PF significantly increased the serum levels of IgG subclasses, as well as T lymphocyte proliferation, when administered in pigs with an inactivated vaccine against PPV. The enhanced IgG subclass levels paralleled the increased serum levels of IFN-γ and IL-2. This adjuvant activity was evident in the increase in both cellular and humoral immune responses. In addition, PF could inhibit PPV-induced apoptosis by immune enhancement. Interestingly, FA as a PF component's significance inhibited the PPV replication. Also, FA is a potential antiviral adjuvant, which could be widely used in veterinary clinics.

## Data Availability Statement

The original contributions presented in the study are included in the article/supplementary material, further inquiries can be directed to the corresponding authors.

## Ethics Statement

The animal study was reviewed and approved by Binzhou Animal Science and Veterinary Medicine Academy, Shandong province (NO. SYXK (lu) 20110066). Written informed consent was obtained from the owners for the participation of their animals in this study.

## Author Contributions

XM and ZZ: research concept, methodology, data extraction, analysis, and draft writing. ZG, YLi, and LZ: resource searching, verification, formal analysis, supervision, and manuscript reviewing and editing. KY and XL: resources, methodology, project administration, supervision, and manuscript reviewing and editing. YLiu and ZS: resource searching and manuscript reviewing and editing. XM and ZZ: methodology and manuscript reviewing and editing. All authors contributed to the article and approved the submitted version.

## Funding

The present work was funded by the National Natural Science Foundation of China (Grant nos. 31602098 and 32072906), the Scientific and Technological Project of Henan Province (Grant nos. 192102110188, 192102110184, and 212102110103), the Postgraduate Research and Practice Innovation Program of Jiangsu Province (KYCX20-1494), the veterinary drugs science subject in Henan University of Animal Husbandry and Economy (Grant no. 41000003), the scientific research and innovation team in the Henan University of Animal Husbandry and Economy (Grant no. 2018KYTD18), and the science and technology major project of Prevention and Treatment of Major Infectious Diseases such as AIDS and Viral Hepatitis: Research on new technology of integrated field rapid detection of important viruses (Grant no. ZX10711001-003-003).

## Conflict of Interest

The authors declare that the research was conducted in the absence of any commercial or financial relationships that could be construed as a potential conflict of interest.

## Publisher's Note

All claims expressed in this article are solely those of the authors and do not necessarily represent those of their affiliated organizations, or those of the publisher, the editors and the reviewers. Any product that may be evaluated in this article, or claim that may be made by its manufacturer, is not guaranteed or endorsed by the publisher.

## Abbreviations

BC: blank control group
CCK-8: cell counting kit-8
DMEM: Dulbecco's modified eagle medium
ELISA: enzyme-linked immunosorbent assay
HA: hemagglutination
HI: hemagglutination inhibition
IgA: immunoglobulin A
IgG: immunoglobulin G
IgM: immunoglobulin M
IL-2: interleukin 2
IL-4: interleukin 4
IL-6: interleukin 6
IL-10: interleukin 10
IL-12: interleukin 12
IFN-γ: interferon-γ
MTT: methyl thiazolyl tetrazolium
OA: oil emulsion adjuvant
PA: propolis flavonoid adjuvant
PBS: phosphate-buffered saline
PF: propolis flavonoid
PPV: porcine parvovirus
UPLC-Q/TOF-MS: ultra-performance liquid chromatography-quadrupole/time-of-flight mass spectrometry.
